# Impact of Health System Inputs on Health Outcome: A Multilevel Longitudinal Analysis of Botswana National Antiretroviral Program (2002-2013)

**DOI:** 10.1371/journal.pone.0160206

**Published:** 2016-08-04

**Authors:** Mansoor Farahani, Natalie Price, Shenaaz El-Halabi, Naledi Mlaudzi, Koona Keapoletswe, Refeletswe Lebelonyane, Ernest Benny Fetogang, Tony Chebani, Poloko Kebaabetswe, Tiny Masupe, Keba Gabaake, Andrew F. Auld, Oathokwa Nkomazana, Richard Marlink

**Affiliations:** 1 Harvard T.H. Chan School of Public Health, Boston, MA, United States of America; 2 Ministry of Health, Gaborone, Botswana; 3 University of Botswana, Gaborone, Botswana; 4 Division of Global HIV/AIDS, Center for Global Health, U.S. Centers for Disease Control and Prevention, Atlanta, Georgia, United States of America; University of Malaya, MALAYSIA

## Abstract

**Objective:**

To measure the association between the number of doctors, nurses and hospital beds per 10,000 people and individual HIV-infected patient outcomes in Botswana.

**Design:**

Analysis of routinely collected longitudinal data from 97,627 patients who received ART through the Botswana National HIV/AIDS Treatment Program across all 24 health districts from 2002 to 2013. Doctors, nurses, and hospital bed density data at district-level were collected from various sources.

**Methods:**

A multilevel, longitudinal analysis method was used to analyze the data at both patient- and district-level simultaneously to measure the impact of the health system input at district-level on probability of death or loss-to-follow-up (LTFU) at the individual level. A marginal structural model was used to account for LTFU over time.

**Results:**

Increasing doctor density from one doctor to two doctors per 10,000 population decreased the predicted probability of death for each patient by 27%. Nurse density changes from 20 nurses to 25 nurses decreased the predicted probability of death by 28%. Nine percent decrease was noted in predicted mortality of an individual in the Masa program for every five hospital bed density increase.

**Conclusion:**

Considerable variation was observed in doctors, nurses, and hospital bed density across health districts. Predictive margins of mortality and LTFU were inversely correlated with doctor, nurse and hospital bed density. The doctor density had much greater impact than nurse or bed density on mortality or LTFU of individual patients. While long-term investment in training more healthcare professionals should be made, redistribution of available doctors and nurses can be a feasible solution in the short term.

## Introduction

The global campaign against the HIV/AIDS epidemic has had such significant achievements that UNAIDS is now embarking on a Fast-Track strategy to end the AIDS epidemic by 2030 [1]. However, one of the challenges to achieving this goal is mobilization of the essential resources this strategy needs. This challenge will be quite similar to those faced by the scale-ups of antiretroviral therapy (ART) program in the last decade.

ART scale-up needed a multitude of experienced and trained health professional [2]. The human resource capacity in sub-Saharan Africa was so low that some had suggested that ART programs scale-up would fail on that account [3,4]. A good example of a successful scale-up effort is Botswana. The health system of this country had to cope with the tremendous burden of the HIV epidemic, despite low health worker to population ratios and insufficient infrastructure [5,6].

Identified as the country with the second-highest HIV prevalence in the world after Swaziland [7], Botswana embarked upon a large scale antiretroviral treatment (ART) program in 2002. By the end of 2014, the number of people enrolled across the country in the National ART Program, called Masa, Setswana word for “a new dawn,” had reached 247,856, a 71-fold increase from 3,500 in 2002 [8]. This massive influx of patients, who needed a wide variety of services, put considerable pressure on the health system of this country [9], because the health professionals had not only to provide care for the HIV-related conditions, but also to deal with the significant burden of non-communicable diseases both among HIV and general population in Botswana [10].

At the outset of the Masa program, Botswana, like other sub-Saharan countries, faced a human resource crisis [11,12]. The country had low physician to patient ratios. However it should be noted that Botswana, in comparison to other countries in the region, enjoys a significantly higher ratio of health professionals to population. For example, according to the Global Health Observatory of the World Health Organization, between 2005 and 2012 on average there were 3.4 doctors per 10,000 Batswana people. This is well below the world average, 13.9, but much above countries like Zimbabwe and Tanzania, with 0.6 and 0.1 doctors per 10,000 population, respectively. A similar pattern is seen with regards to nurses in the same time period. In Botswana, there were 28.4 nurses per 10,000 population, while in Zimbabwe and Tanzania, there were only 12.5 and 2.4 nurses per 10,000 population, respectively [13].

Botswana’s healthcare delivery system, based on a doctor-care model, had to deal with not only inadequate number of health professionals but with their maldistribution and high turnover [14]. The challenge was not only the scarcity of all cadres of trained health-care workers, because of government economic and fiscal constraints, but also the scarcity of training mechanisms to meet the demands of the HIV/AIDS epidemic. To address the shortage of doctors, while increasing accessibility to ART, in 2006 the Ministry of Health in collaboration with its national and international partners started training nurses in dispensing and prescribing antiretroviral (ARV) drugs to stable patients [5]. As part of the training, the nurses learn to correctly prescribe ARVs, to identify and appropriately manage adverse reactions related to ARVs, to address adherence issues, and to understand when referral is needed (e.g., virological failure, severe toxicity). By 2010, more than 200 nurses were trained to prescribe and 800 nurses were trained to dispense ARVs. However, because of many logistical and financial challenges, only a limited number of dispensing and prescribing nurses were trained afterwards.

Our previous analysis of the Botswana national ART program showed significant reduction in mortality among HIV-infected individuals receiving ART, to levels much the same as those reported by other low-income or middle-income countries [8]. The program also reduced mortality in the first year of treatment initiation; the high initial mortality after treatment initiation shows the importance of the change in the national guidelines, which requires earlier treatment, from CD4 cell counts below 200 cells/μL in 2002, to below 250 cells/μL in 2008, and to below 350 cells/μL in 2013. That analysis also found that loss to follow-up, or lack of electronic documentation, to be an important issue to be addressed, because some researchers suggest that retention of patients might be a good overall indicator of program effectiveness [15].

Currently, the Ministry of Health of Botswana, in line with the UNAIDS Fast-Track strategy is evaluating different prevention and treatment models. One of these models under review is “test and treat,” which offers hope for improving the care of HIV-infected people and preventing transmission of the disease. This treatment model, i.e., ART initiation regardless of CD4 cell count and clinical status, can have significant effect on HIV epidemic trajectory. However, its implementation will pose new challenges to the healthcare delivery system as more and more patients will need ART.

To inform this policy and to help develop a strategic plan, there is an urgent need to assess the performance of the Masa Program. At this part of our assessment, we focused on one aspect of health (mortality reduction) and its correlation with certain resources in the healthcare delivery system. Specifically, this study aimed to measure the extent to which health system resources, i.e., the human resources for health and infrastructure have contributed to reductions in mortality among HIV-infected individuals in the Masa Program. It was designed to exploit the strengths of a relatively rich dataset collected by Masa Program across the districts over 12 successive years.

## Methods

### Data sources

Two main categories of data were used in the analysis: patients’ clinical records and district-level health system resources data, e.g., human resources and number of hospital beds. The clinical data are from the routinely gathered information in 155 facilities across 24 districts involved in the program and include de-identified data on demographics, ART initiation date, CD4 cell counts, drug regimens, and mortality, from January 2002 through October 2013. Details of the database have previously been described [8].

In this analysis we analyzed the data for 97,634 HIV-infected adult (18 years and above) who received ART in the National Program. We had to remove 85,538 individuals whose ARV regimen was unknown and 28,057 with no CD4 cell count and 3,468 individuals who discontinue their HIV treatment after the first visit.

The database for health system resources were compiled from several sources. The Botswana Central Statistics Office had conducted three rounds of the Botswana Health Facility Survey (HFS) in 2006, 2008 and 2009. Our study team implemented another survey round in 2014 to acquire the most recent set of observations for the longitudinal analysis. Using the four rounds of HFS data in a linear regression model, we estimated the number of resources in the years where districts’ data were not available. We also utilized databases collected by the U.S. President's Emergency Plan for AIDS Relief (PEPFAR) Master Trainers and KITSO training programs to adjust our estimates. Master Trainers Program was a PEPFAR funded training program for doctors, nurses and lab technicians. It began in 2004 and was aimed at developing sustainable training capacity in clinical care and treatment of HIV/AIDS patients. "Kitso" is the Setswana word for "knowledge." KITSO AIDS also stands for "Knowledge, Innovation, and Training Shall Overcome AIDS." The KITSO AIDS Training Program was a standardized training in HIV and AIDS care, developed specifically for Botswana’s health professionals. Since 2002, KITSO has trained over 7,000 healthcare workers, including doctors, nurses, midwives and pharmacists. The World Health Organization (WHO) dataset on human resources for health was used to cross-check the total number of physicians, nurses and hospital beds estimated for each year [16]. Population data were taken from the Botswana Census in 2001 and 2011 and census projections. The census projections were cross-checked with the data from the Botswana AIDS Impact Survey, a nationally representative survey conducted in 2004, 2008 and 2013. Health system resource density was defined as the number of doctors, nurses and hospital beds per 10,000 population in each health district in each year. The data from the above sources were merged into a single dataset and organized into a longitudinal (person-quarter) format. We assumed that doctors, nurses, hospital densities remained constant in each year.

### Data analysis

The outcome of interest is binary response (dead or not) or (LTFU or not). We used marginal structural modeling (MSM) to determine the effects of health system resources on mortality during ART while accounting for the individual-level variables [17]. We used MSM because use of standard regression models for the analysis of cohort studies with time-dependent covariates may results in biased estimates [18]. To estimate the model, we first estimated the inverse probability weights (IPWs) of being treated; and then used these weights in the final regression model. IPWs were estimated by combining the inverse-probability-of-treatment weights (IPTW) and inverse-probability-of-censoring weights (IPCW), which accounted for informative censoring from receiving treatment. Using observed variables, this approach allowed us to derive a weight for each individual in each quarter, i.e., the inverse probability of remaining in the study in that interval [19]. In other words, this weight is intended to account for the censoring due to LTFU for each individual in each quarter. The stabilized version of IPTW and IPCW were estimated as a ratio of two predicted probabilities from two logistic regression models. The model for the numerator of this ratio includes only the baseline covariates, while the denominator includes baseline and time-varying covariates [20]. To estimate the model, we used pooled logistic regression with an interval-specific intercept, modeled by restricted cubic splines with knots at the 10th, 50th, and 90th percentiles [20].

The explanatory variables at individual level included age (= 1 if 40 years or older) and gender, CD4 (= 1 if CD4 cell count is more than 100), ARV regimen, and year of treatment initiation; and at district level included hospital bed, doctor and nurse density. Because socio-economic factors, such as poverty or level of education in each district could be potentially correlated with mortality or LTFU in that district, and the yearly data for each district during the study period were not available, we included an indicator variable for each district as fixed effects in the model to capture district heterogeneity. To correct for auto-correlation in the panel setting as well as patients clustered at the district level, we used the method developed by Cameron, Gelbach and Miller to account for clustering at multiple dimensions at the same time [21].

After treatment initiation in the Masa Program, each patient was expected to have a clinic visit every six month. However, there were lab tests and prescription refills between visits. Because of that, the dataset was organized by quarters. If there were more than one CD4 cell count in a quarter, the mean value was used. District-level variables included hospital bed, doctor and nurse density. We tested different specifications for years of treatment initiation such as continuous (both linear and quadratic), and indicator variables for each year and binary variable (before vs after 2008). The year 2008 was selected as the new guidelines came into effect. According to the new guidelines CD4 eligibility for ART initiation became 250, up from 200 CD4 cells/mm^3^ and zidovudine was replaced by tenofovir in the first line of treatment. We found the binary the best fit for the model. We also found that quadratic functions of densities were the best options for the model. We used the Hosmer—Lemeshow goodness-of-fit test, as well as Akaike information criterion (AIC) and Bayesian information criterion (BIC) for our model selection. We used Gaborone, the capital with the main referral hospital, as the reference district. The assumption was made that human resources and hospital bed density was proportionate to the availability and distribution of other resources and thus, could serve as a proxy for overall availability of health services.

The results are presented graphically as predicted probabilities of mortality or LTFU, adjusted by all the independent variables in the model [22]. All analyses were done with STATA/MP 14.1 software [23].

The study was approved by Harvard School of Public Health IRB and Botswana’s Health Research and Development Committee (HRDC). Individual consent was not required by either IRB as the analysis was performed on de-identified data sets. There is no conflict of interest that could be perceived as prejudicing the impartiality of the research reported.

## Results

### Descriptive statistics

#### Patient-level

We analyzed the association between health system inputs and health outcome of 97,627 HIV-infected adult patients across 24 health districts in Botswana from January 2002 through October 2013. Table 1 contains descriptive statistics for the patients in the Masa program. The majority of the study population was female (61,176 women [62.7%]), and the median age at ART initiation was 33 years for women (interquartile range [IQR] 28–40) and 38 for men (IQR 32–45). In this analysis, 3,270 (5.4%) women and 3,278 (9.0%) men died. The median baseline CD4 cell count for men was 120 cells/ mm3 (IQR 60–179), and that of women was 150 cells/ mm3 (IQR 86–196). The overall rate of LTFU was 12.47 per 100 P-Y (95% CI 12.38–12.55). There was a great range of overall LTFU across districts, including rates as low as 4.6 losses per 100 P-Y in Kgalagadi North, and 5.9 losses per 100 P-Y in Kgalagadi South, to rates as high as 25.4 losses per 100 P-Y in Kweneng East and 46.3 losses per 100 P-Y in Ngamiland. Details of mortality and LTFU variation in the Masa Program have previously been described [24]. Overall median follow-up time was 40 months (IQR 15–79). The median duration of follow-up for patients who were LTFU was 27 months (IQR 12–49), compared with a median of 64 months (IQR 21–99) for those who were not lost. Zidovudine and tenofovir were the main nucleoside reverse transcriptase inhibitor (NRTI) medications used as the first line of treatment, with 64% of people starting with zidovudine compared to 29% on tenofovir with the remainder of patients, 7%, on stavudine. The population on non-nucleoside reverse transcriptase inhibitors (NNRTIs) was split between efavirenz and nevirapine, 54.3% and 39.4% respectively, with the remainder of patients, 6.3%, starting on the protease inhibitor, ritonavir-boosted lopinavir.

**Table 1:** Baseline Patient Characteristics for Patients† Initiating Antiretroviral Treatment in the Botswana National HIV/AIDS Treatment Program, 2002–2013.

Continuous Variables	Mean	Std. Dev.	Median	IQR
Number of Clinic Visits	10.7	8.2	9	4–15
Months of Follow-up	48.7	39.1	40	15–79
Age	36.3	9.1	35	29–42
Baseline CD4	150.6	117.1	139	75–191
**Indicator Variables**	**No. of Patients**		**% of study population**	
Women	61,173		63	
**Year of Initiation**				
2002–2007	66,602		68	
2008–2013	31,025		32	
**First-line ART regimen (NNRTI or PI)**				
Nevirapine	38,442		39	
Efavirenz	53,074		54	
Lopinavir	6,111		6	
**First-line ART regimen (NRTI)**				
Zidovudine	62,508		64	
Stavudine	7,196		7	
Tenofovir	27,923		29	

^†^Table 1 reports on the 97,627 patients included in the regression model.

### District-level

**Fig 1:**
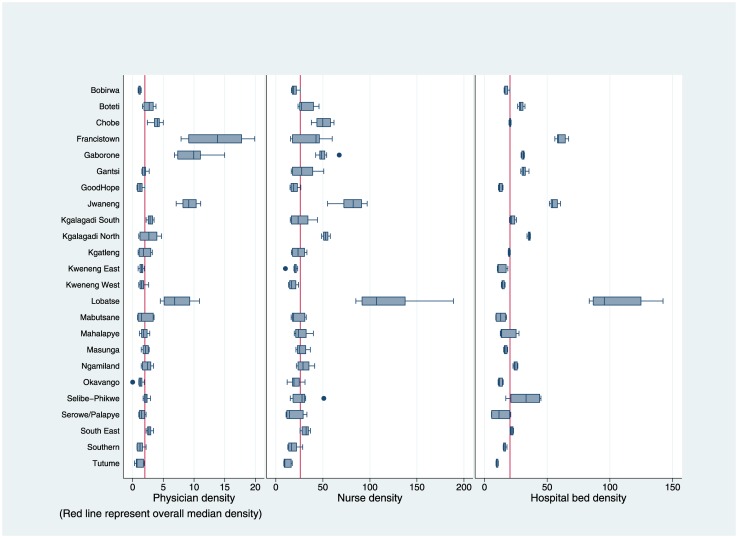
Physician, Nurse, and Hospital Bed Density per 10,000 Population in Botswana Health Districts (2002–2013).

Average doctor density across districts was widely distributed (Fig 1). Median doctor density in 2013 was 2.7, up from 1.5 in 2006 (Table 2). Lobatse, with the only mental institution in Botswana and Jwaneng with the richest diamond mine in the world are considered outliers. Twenty districts had fewer than four doctors per 10,000 population on average over the study period, with the remaining four having doctor densities ranging between 7.2 and 13.6 doctors. Across years, doctor density in many districts showed modest increases, with a few notable exceptions. Gaborone experienced a decrease in doctor density over time, from 10.5 in 2006 to 8.1 in 2013. Conversely, Lobatse, Kgatleng, Mabutsane, and Kgalagadi North have seen significant increase in doctor density over these years.

**Table 2:** Distribution of physicians, nurses and hospital beds in selected years at Botswana health districts.

	Physician density			Nurse density			Hospital bed density		
District name	2006	2009	2013	2006	2009	2013	2006	2009	2013
**Bobirwa**	0.9	1	1.4	18.4	20.4	17.6	16.7	16.1	20.2
**Boteti**	1.7	3.3	3.3	26.3	39.2	27.2	29.1	27.7	32.1
**Chobe**	3.5	4.2	4.3	60	43	37.9	20.9	19.6	20.8
**Francistown**	8.6	17.4	16.5	46.1	15.7	46.3	59.7	58.8	66.8
**Gaborone**	10.5	6.8	8.1	52.4	44.7	67.4	31.5	29.2	31.7
**Gantsi**	1.9	1.5	2	18.3	38.3	51	30.8	31.4	32.5
**GoodHope**	0.8	1.4	2	16.8	22.5	21.7	12	12.2	14.3
**Jwaneng**	7.8	9	10.5	94.3	69.2	55	55.2	52.9	60.3
**Kgalagadi**	2.4	3	3	17.1	29.9	44.2	21.2	21.7	25.4
**Kgalagadi North**	1.1	3.7	4.7	56.2	50.3	55.4	36.4	33.9	36.1
**Kgatleng**	1	2.9	2.6	18.4	28.3	32.2	20.1	18.9	20.5
**Kweneng East**	1.2	1.6	1.9	21.4	21.3	23.3	11.1	17.3	17.3
**Kweneng West**	1.1	1.7	2.6	15.2	20.8	19.3	14.3	14.3	16.4
**Lobatse**	4.9	7.8	10.8	94.9	134.8	116	88.9	102.3	141.8
**Mabutsane**	0.9	3.3	3.5	18.2	30.2	32.3	9.6	16.3	16.9
**Mahalapye**	1.4	2.2	2.4	21.9	31.6	25.9	13.8	21.9	27.4
**Masunga**	1.7	2.4	2.7	23.8	29.9	36.7	16.3	16.6	18.4
**Ngamiland**	1.5	2.7	3.1	24.4	32.7	41.3	24.2	24.4	26.5
**Okavango**	1	1.3	1.9	19	23.2	31	11.7	13	14.9
**Selibe-Phikwe**	2	1.7	2.9	30.9	15.4	51	45.1	17.5	23.9
**Serowe/Palapye**	1.1	1.9	1.7	12.1	28.4	16.5	5.9	18.2	20.9
**South East**	2.4	3	2.6	35.7	27.5	31.1	23.1	21.5	20.8
**Southern**	0.7	1.4	2.2	14.2	18.8	28.6	16	15	17.9
**Tutume**	0.6	1.6	1.9	9.5	15.3	16.9	9.8	9.8	11.1
**National mean**	2.5	3.6	4.1	31.9	34.6	38.6	26.0	26.3	30.6
**National median**	1.5	2.6	2.7	21.7	29.2	32.3	20.5	19.3	20.9

Densities are calculated for every 10,000 people in the district in that year. Data Sources: 2006 and 2009 from The Botswana Central Statistics Office, 2013 data was collected by the study team.

Nurse density had a similar pattern to doctor density (Fig 1). Median nurse density in 2013 across districts was 32.3 nurses per 10,000 population, up from 21.7 in 2006 (Table 2). Between 2002 and 2013, average nurse density across health districts ranged from 12.1 per 10,000 population in Tutume to 119.8 nurses per 10,000 population in Lobatse. Sixteen districts had average nurse densities of fewer than 30 nurses per 10,000 population. In 2013 Botswana’s two largest cities Gaborone and Francistown had nurse density of 67.4 and 46.3, respectively. Their respective neighboring districts Good Hope (130 Km south of Gaborone) and Bobirwa (230 Km from Francistown) had average nurse density of 21.7 and 17.6 nurses per population. Over time, Chobe and Jwaneng experienced noticeable decreases in nurse density, while Gantsi, Kgalagadi, Selibe-Phikwe showed considerable gains in nurse density.

Average hospital bed density was correlated with population density, with a correlation of 0.59. Median hospital bed density rose slightly from 20.5 beds in 2006 to 20.9 in 2013. The lowest average bed density was in Tutume with 10 beds per 10,000 population, while the highest was Lobatse with 104.9 beds per 10,000 population. In 2013 Francistown had the second highest hospital bed density with 66.8 beds per 10,000 population, while Gaborone only has 31.7 beds per 10,000 population. Both Gantsi and Kgalagadi North, which are rural districts, had higher hospital bed density than Gaborone. In 2013 hospital bed density in eight districts was less than 20 beds per 10,000 population. Over time bed density in Selibe-Phikwe was noticeably decreased, while it was substantially increased in Lobatse, Mahalapye and Serowe/Palapye. Other districts experienced modest changes in bed density over this time period.

The indicator variables for the health districts, which were intended to capture unmeasured district-level heterogeneity such as poverty and level of education of the population, were highly significant. Variation in mortality and LTFU among HIV-infected Batswana at district-level have been discussed somewhere else [24].

### Regression analysis

**Fig 2:**
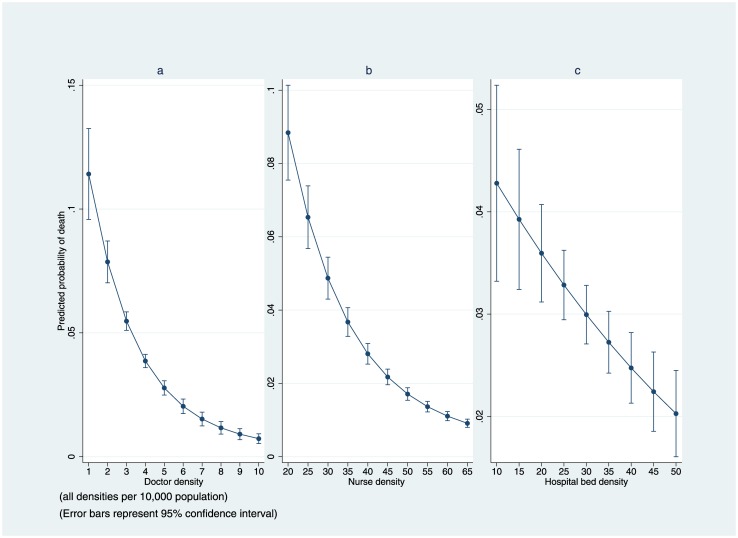
Adjusted Predictions of Mortality in Masa Program (2002–2013).

**Fig 3:**
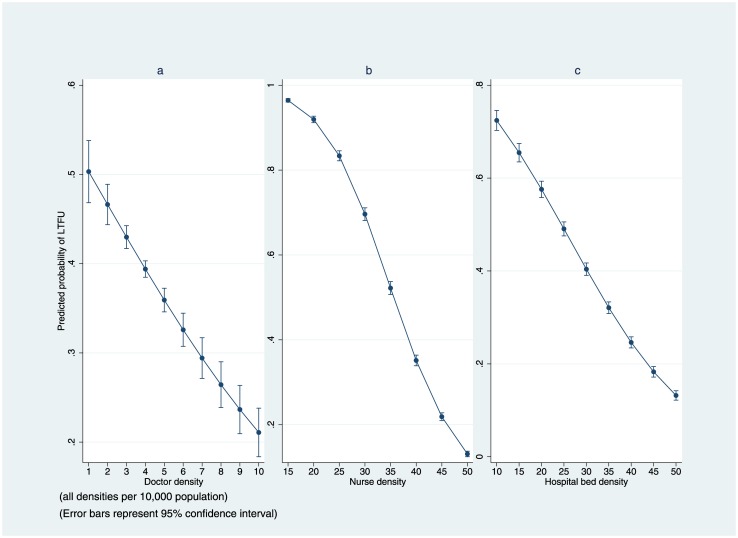
Adjusted Predictions of LTFU in Masa Program (2002–2013).

The results of the multilevel logistic regression are provided in Table 3. Holding individual patient’s characteristics constant, doctor, nurse and hospital bed density had a significant effect on the mortality of patients across districts. Fig 2a, 2b and 2c as well as Fig 3, 3b and 3c illustrate the adjusted predicted probability of mortality or LTFU, which were nonlinearly correlated with doctor, nurse and hospital bed density. These figures show that as doctors, nurses and hospital bed density increases, the probability of death (or LTFU) at individual level decreases. Increasing doctor or nurse density was much more impactful on the probability of mortality at lower densities; but the effect diminishes as doctor or nurse density approaches higher levels. For instance, when doctor density changes from one doctor per 10,000 population to two doctors, the predicted probability of death for each person drops by 27%, from 0.11 (95% CI 0.10–0.13) to 0.08 (95% CI 0.07–0.9), holding all other covariates at mean value (Fig 2a). However, when doctor density changes from four doctors per 10,000 population to five doctors, the predicted probability of death for each individual patient decreases 25%, from 0.04 (95% CI 0.038–0.044) to 0.03 (95% CI 0.026–0.033), holding all other covariates at mean value. We assessed various interactions between district-level and individual-level variables; none of them was statistically significant.

**Table 3:** Multilevel Analysis of Variables Predictive of Overall Survival and LTFU among Adult Patients in Masa Program.

	Mortality		LTFU	
Variables	Adjusted OR	95% CI	Adjusted OR	95% CI
**District-Level**				
Physician density	0.7***	[0.60,0.71]	0.9***	[0.82,0.93]
Physician density Squared	1.0***	[1.0,1.01]	1.0	[0.99,1.0]
Nurse density	0.9***	[0.91,0.93]	0.8***	[0.81,0.82]
Nurse density Squared	1.0***	[1.0,1.0]	1.0***	[1.0,1.0]
Hospital bed density	0.99	[0.98,1.0]	0.94***	[0.94,0.95]
Hospital bed density Squared	1.0**	[1.0,1.0]	1.0***	[1.0,1.0]
**Individual-Level**				
Baseline CD4				
0–49	1	[1,1]	1	[1,1]
50–249	0.5***	[0.47,0.53]	1.1***	[1.05,1.15]
250–499	0.44***	[0.38,0.50]	0.77***	[0.71,0.84]
500+	0.3***	[0.22,0.41]	1.27**	[1.1,1.48]
NRTI				
Tenofovir	1	[1,1]	1	[1,1]
Zidovudine	1.34***	[1.23,1.46]	2.51***	[2.39,2.64]
Stavudine	5.38***	[4.8,5.99]	2.3***	[2.14,2.51]
NNRTI				
Nevirapine	1	[1,1]	1	[1,1]
Efavirenz	1.20***	[1.11,1.29]	0.92***	[0.88,0.96]
Lopinavir	0.64***	[0.55,0.75]	0.69***	[0.64,0.75]
Initiation Year <2008	1	[1,1]	1	[1,1]
Initiation Year >2008	0.91*	[0.84,0.99]	0.56***	[0.53,0.59]
Sex				
Male	1	[1,1]	1	[1,1]
Female	0.66***	[0.62,0.71]	0.95*	[0.91,0.99]
Age category				
Age < = 40	1	[1,1]	1	[1,1]
Age > 40	1.31***	[1.23,1.39]	0.86***	[0.82,0.89]

Densities are calculated for every 10,000 people in the district in that year. For example, physician density (per 1 doctor increase for every 10,000 population). Level of statistical significance: * p<0.05, ** p<0.01, *** p<0.001. Models are run with health district fixed effects (not reported here). Models are weighted to account for LTFU.

A similar pattern was seen when we predicted the probability of death at different levels of nurse density. For example, when nurse density increased from 20 nurses per 10,000 population to 25 nurses, the predicted probability of death for each patient dropped 28%, from 0.09 (95% CI 0.08–0.10) to 0.065 (95% CI 0.06–0.07), holding all other covariates at mean value (Fig 2b). However, when nurse density changed from 35 nurses per 10,000 population to 40, the predicted probability of death for each patient decreased 25%, from 0.04 (95% CI 0.033–0.041) to 0.03 (95% CI 0.025–0.031), holding all other covariates at mean value. Adjusted predictions of mortality were inversely related to hospital bed density. A nine percent decrease was noted in predicted mortality of an individual in the Masa program for every five hospital bed density per 10,000 population increase.

The same procedures followed for mortality (Fig 2a, 2b and 2c) were followed to plot the predicted probabilities for LTFU (Fig 3a, 3b and 3c). The pattern of predicted probability of LTFU, however, was somewhat different. Fig 3a represents linear changes of predicted probability of LTFU over different values of doctor density. Accordingly, by increasing one doctor per 10,000 population, probability of a patient in the Masa program becoming LTFU decreases by eight percent, holding all the other covariates at their mean value. The effect of nurse density on predicted probability of LTFU follows an inverse sigmoid curve (Fig 3b), with the steepest part of the slope between 30 and 35 nurses per 10,000 population (spanning national median [32.3] in 2013), with 26% decreases in probability of LTFU, from 0.70 (95% CI 0.68–0.71) to 0.52 (95% CI 0.51–0.54). As for the hospital bed density, every five additional beds per 10,000 population reduced the probability of LTFU for an individual patient by almost 14 percent.

## Discussion

This study is the first multi-level longitudinal analysis of the effect of health system inputs at district-levels on probability of death at individual-level among HIV-infected individuals receiving ART. Predictive margins of mortality and LTFU were inversely correlated with doctor, nurse and hospital bed density. The number of the patients in the Masa program increased more than 70-fold from 2002 to 2013, yet the health system resources did not keep up at the same pace. Resources (health-workforce and hospital beds) increased across the districts between 2002 and 2013, nonetheless much less than the system’s need [25].

The results of this study indicate that doctor density had much greater impact than nurse or bed density in Botswana. This is consistent with the doctor-centered approach to care and the limited task-shifting to nurses described. This means that the availability of certain types of healthcare worker is important, in addition to the overall density of healthcare workers, in determining district-level mortality or LTFU. Healthcare delivery may also need some revision. There is strong evidence indicating the feasibility and effectiveness of task-shifting strategy in sub-Saharan countries [26]. Botswana has already moved toward task-shifting by training nurse prescribers and nurse dispensers and designed simplified ART guidelines to delegate a number of tasks from medical doctors to nurses. Expansion of task-shifting can be part of the solution [27].

Botswana, like other sub-Saharan African countries, has the lowest doctor and nurse to population density in the world [28]. Inadequate capacity to train and retain professional health workers in the Botswana public sector make the picture all the more complicated. Most of the nurses (84%) in the country are Botswana citizens who have been trained in national institutions. However, only 21% of the doctors registered with Botswana Health Professions Council by October 2012 were Batswana, and all were trained outside of the country [14]. The Government has to attract both expatriate doctors and Botswana doctors being trained in 21 countries in Europe, North America, South America, Australia, Africa and South East Asia [14]. Doctors trained in various countries may find it difficult to adjust to a single model of care, with limited level of resources, which in turn could affect the quality of care, and the care continuum. However, lack of longitudinal data on doctors training background prevented us from further analysis. The critical shortage of health personnel was further complicated by inequality in geographic distribution.

We found significant variation across health districts with regards to the distribution of health system resources. While the ultimate goal of the health system should be equitable distribution of resources, our findings indicate that allocating more resources to the worst-off districts will be most efficient. The modest increase in health system resources over the last decade could have been much more effective if the district with lower density had received a bigger share, Training more doctors and nurses should be a long-term solution. Meanwhile, redistribution of available doctors and nurses can be a feasible solution in the short term.

Comparatively, the weakest association was between infrastructure (reflected by hospital bed density in this analysis) and patient-level mortality or LTFU. However, this finding should not diminish the importance of infrastructure in healthcare delivery. Considering the fact that median hospital bed density rose from 20.5 beds in 2006 to 20.9 in 2013, more investment in infrastructure may be needed. Nonetheless, a recent study in Botswana shows that because of maldistribution, some districts have significant overcapacity while some others under considerable need for hospital beds [6].

### Limitations

There are limitations to this study that should be acknowledged. The main limitation is that the patient’s cause of death is unknown. Therefore, it is all-cause mortality analysis. Some of the patients may have died of non-AIDS related causes. Another limitation is the potential for missing death data due to inconsistent reporting. In this analysis we could obviously use only those deaths recorded in the electronic database of the National Program. However, deaths might not be reported to HIV clinic staff and non-institutional deaths might not be reported to the health-care system as is legally required. Therefore, LTFU might include patients who died without a death report captured in the database. Two systematic reviews showed that, at 2 years, African ART programs retain only about 60% of their patients, largely because of high LTFU rates [29,30]. Using a method to trace patients, a study of 524 patients in Botswana in early 2003 showed that more than half of the 68 patients originally deemed to be lost to follow-up were confirmed to be dead after tracing. Therefore, many of the patients who are LTFU in our study have probably died [31].

Some of the LTFU as well as missing data for ART regimen and CD4 cell count can be explained by the poor quality of data reporting concerning all the patients at certain facilities for a period of time, rather than a selective process affecting a particular group of patients, or some of the facilities in certain districts. There was a significant variation in data quality across the districts and facilities in Botswana. Some facilities reported only demographic data and failed to report the laboratory results or prescription drug regimens of clients. Even the reporting, or lack thereof, was not consistent, and fluctuated over time. Facility’s failure to enter the data was mainly due to understaffing, inadequate training, and lack of infrastructure in general. Improving the data collection process should be a priority in the Masa Program. We tried to account for this through our analysis of patients lost to follow-up, specifically in the regression model, by using marginal structural modelling to correct for bias that it may have introduced. There was also a meaningful direct correlation between documentation and other resources, e.g., doctors, nurses and hospital beds in the facilities. Therefore, by including those variables as proxy for health system resources along with the indicator variable for the districts as fixed, we have not introduced selection bias to this analysis, or have limited the generalizability of these findings.

A further limitation was the absence of training data for nurses. As number of nurses trained to prescribe or dispense ARV has been increasing over these years, including density of prescribing and dispensing nurses rather than a general category for nurses, could show stronger effect for these subgroups of nurses. As for the health workforce and hospital bed data we had to estimate the data for several years because of limited rounds of health facility census data. However, combining several databases provided us with the best estimate of the reality in those years. Strengths of this study include our large sample size and repeated measures over 12 years of the Masa Program.

In conclusion, Botswana has successfully scaled up its ART program, providing care to hundreds of thousands of its HIV infected citizens [32]. We found significant relationships between doctor, nurse and bed density and individual mortality or LTFU in Botswana National ART Program in a model that incorporated individual- and district-level variables as well as district fixed effects for unmeasured factors. The findings of our longitudinal subnational study support the results of both sub-national and cross-national studies exploring the health-system inputs and their relation to health outcome, emphasizing the importance of healthcare professionals in the fight against HIV [33,34]. Marked differences in distribution of resources in Botswana districts were translated to significant differences in likelihood of mortality and LTFU. While Botswana should make long-term investment in training more healthcare professionals and in building more healthcare facilities, redistribution of available doctors and nurses can be a desirable solution in the short term, however, the feasibility of this redistribution requires further investigation. Overall, as ARV programs have been scaled up and many HIV-infected individuals receive lifetime care, more efforts should be put into addressing inequities and inefficiencies.
